# The Relation of Insanity to Civilisation—I

**Published:** 1906-08-25

**Authors:** 


### HOSPITAL ADMINISTRATION.

### CONSTRUCTION AND ECONOMICS.

### THE RELATION OF INSANITY TO CIVILISATION.—I.

Among the moral causes of insanity the passions must be reckoned the chief; their action is visible every instant. In some men it is self-love, vanity, ambition, or pride that exalts and deranges the intellect, in others it is jealousy, religious or political fanaticism, or love. Reverses of fortune, vexation, the obstinate struggles of moral principle with desires often irresistible, the obstacles that men meet with when trying to effect their schemes, and in our times scepticism, an affectation of individuality, wildness of commercial speculation, and intemperance are among the most powerful levers. To these must be added avarice, rivalry, hatred, and the thirst for vengeance, want, misery, and despair.

What stronger proofs can be cited in support of the position that moral causes predominate over physical than the numerous instances of insanity that bear the impress of each epoch and each country? The political crises that from time to time shake the foundation of social order, those remarkable events that are the distinctive and salient features of a century, belong not to history alone; they are within the territory of medical science, and from them an exact list of human aberrations might be drawn up. Thus, when some great catastrophe has caused the death or ruin of a great number of men, the apparent victims do not alone indicate the extent of the disaster. The rebound is felt afar off and rings in the minds of a multitude of feeble spirits. There is, in fact, a considerable mass of individuals floating on the surface of society doomed to insanity by their organisation. Weak of mind, the issue of parents themselves affected with mental disease, eccentric, passionate, and fantastical, they receive, like softened wax, every impression from without; and their reason, vitiated from improper education and a defective state of the organs, cannot withstand any shocks. They are easily to be recognised by their faces, their gestures, and their discourse; their physiognomy is inconstant, their conversation jerking and abrupt. Their excessive and groundless gaiety, the rapid succession of their ideas, the ease with which they form and abandon a hundred schemes, their irritability, the wandering of their eyes, the oddness of their conduct, certain disorderly movements, the levity and weakness of their thoughts, and the want of connection and force in their reasoning are the pathological symptoms that presage their future destiny.

The influence of dominant ideas is really astonishing. Thus we learn that certain religious festivals in Greece, and the mysteries of Bacchus in particular, produced the most strange disorders of the mind. The excesses in which the Athenian Thyads indulged will surprise no one who understands how easy it is to excite the lively and ardent imaginations of women. They have been seen more than once to rush in torrents through cities and provinces, half naked, with hair dishevelled, and filling the air with most frightful howls. Some of them, seized suddenly with a species of vertigo, have believed themselves animated by Divine inspiration, and have communicated their frenzied transports to their companions; and only when the delirium was about to cease could remedies and sacrifices bring their minds back to a state of repose.

In the last years of the Roman Republic, and under the emperors, continual proscriptions, multiplicity of punishments, and tyrannical decrees carried terror into private families, and suicidal mania seized on senators, on knights, and on a crowd of distinguished persons. When discouragement and despair seize on nations unsustained by religious faith, they plunge into suicide because death appears a refuge from ill.

The terror, the desolation, the ruin, and the deaths that followed on the irruption of the barbarians shook the minds of all men with fear; and the historians of those days have preserved some curious details of the forms in which the consequent mental disorder manifested itself. The persecutions which the primitive Christians had to endure enlarged considerably the catalogue of mental alienations. The butcherings, the tortures, the horrors of the circus, and the combats of ferocious animals were not without their effect on the minds of the spectators. Such exhibitions either exalt the imagination to the highest degree or harrow it up with terror; and each of these dispositions is eminently peculiar to insanity. Proneness to imitation, moreover, that true moral contagion, contributed much to the increase of madmen, an account of whose peculiar vagaries will be found in the golden legend and the writings of Baronius Demononomania is one of the most marked types of this period.

In the Middle Ages continual wars, the irruption of barbarians, the profound ignorance of the people and the grandees, and the love of the marvellous which was the consequence of that ignorance, had developed to a degree almost incredible a passion for arms, religious enthusiasm, and a belief in supernatural visitations and agency. Alchemy and judicial astrology were the favourite studies of the strongest intellects. The voice of Peter the Hermit had scarcely been heard when a universal delirium seized on the minds of men; men and women, old men and children, followed in crowds on the footsteps of the monk. Excitement such as this was singularly favourable to mental disorders, and we find the annals of the crusades filled with apparitions of angels and of saints, with Divine revelations and fabulous exploits, and with hallucinations of sight and hearing. Religious and military madness belong chiefly to the first two crusades; in the third the Thanks seem to have abandoned their old character, and to have become imbued with the spirit of romance. The change took place during the reigns of troubadours and knights, who, turning their imaginations towards love and glory, broke out into amorous and chivalrous extravagances. Erotomania, nymphomania, hysteria with its varieties, and the passion for adventure are the distinctive features of this period, and Roland and King Arthur the principal types. With the period of these crusades the sect of political fanatics, called assassins, was contemporaneous. All the works of the time are filled with accounts of their incredible self-devotion, and of the terror which the murders of the many sovereigns that fell under their daggers had inspired. At the signal of their chief, called "the old man of the mountain," they were ready to precipitate themselves from the loftiest towers and to plunge a dagger in their own hearts. So great was their fanaticism that they expired under torments the most cruel without a sigh, regretting only that they had not succeeded. The concomitant excitement of the brain was produced, according to Langles (*Histoire des Croisades*, vol. i.) by an intoxicating drink called *haschischen*, or *has-chitt*, obtained by distillation from the pistils of hemp. Sensual pleasures, moreover, of every kind were employed to perfect that devotion which had commenced in religious education. The eleventh century, celebrated for the first crusade, was witness of a species of insanity that bears much analogy to Tarantism, and is described in the works of Guiopontus, a physician of the school of Salerno, who flourished at that time. During the access of delirium the patients struggled furiously, jumped up into the air with the most savage gestures, wounding themselves and others, and declaring that they heard voices and sounds of different kinds. On hearing the sound of music they gave way to convulsive dancing, until perfectly exhausted by fatigue. These dangerous maniacs were considered to form a part of the legions of Satan.

It is not difficult to explain the maladies that manifested themselves during the period from the twelfth to the fourteenth century. Almost all the countries of Europe had been ravaged by horrible epidemics; measles, smallpox, St. Anthony's fire, and leprosy carried off many persons, and the ravages of the black plague (1380) served to exasperate this mortal suffering.

The sad effects of this last scourge were yet felt, when suddenly a new kind of delirium exhibited itself in Germany and was called St. John's or St. Guy's dance. The individuals attacked danced for hours together, until they fell to the ground deprived of all power. During the attack they were deaf and dumb, except that they bawled out the names of spirits whom they fancied they beheld. Some of them imagined that they were plunged in a stream of blood, which they assigned as a reason for jumping so high; others, again, saw when in their ecstasy, the heavens open, and the Saviour and the Virgin seated on the Throne. It required but a few months to propagate this disease from Aix-la-Chapelle through the Low Countries, where these frenzied dancers appeared with crowns on their heads and linen round the body. As soon, namely, as typanites succeeded to mania, the belly was either bound with linen or the abdomen was cuffed or kicked violently, as was afterwards the practice in cases of convulsions. Labourers quitted the plough, artisans their shops, and mothers of families their children, to join these troops of maniacs. During the fifteenth century exorcism and music were the weapons with which this disease was combated; it diminished in the course of the sixteenth century, and by the end of it had lost all its violence.

Tarantism, which first appeared in Puglia, must be placed in this period. The patients, whether it was that they really had been bitten by a spider or only believed so, fell into a profound melancholy and lost the use of their reason as utterly as if they had been subjected to the influence of intoxication. Some were continually weeping; some were tormented by amorous delirium; and others died under an attack of laughter or despair. As in cases of chorea, the remedy *par excellence*, was music. Tarantism also was propagated by imitation; and it is but justice to say that Paracelsus gives many a warning against moral contagion. Lycanthropy also belongs to this period. The patients imagined themselves to be wolves. In trance they were called *loups garoux*; and the English name is *were-wolf*, though the disease does not appear to have extended to this country. This singular form of madness, which in Russia consigned many to the stake, had its origin in Greece before the Christian era; thence it spread with rapid strides all over Europe, and was transmitted as a sad heritage of antiquity not merely to the peoples of the Roman race, but also to the Germans and Sarmatians.

(*To be continued.*)

